# Changing Clothing

**Published:** 1872-03

**Authors:** 


### CHANGING CLOTHING.

In the latitudes of New England and New York, going westward, the month of March is the most disagreeable of the whole year, with its changing temperature, its slosh and mud, its cold, raw, piercing, damp winds; and although not as cold as January and February, it is more prolific of dangerous diseases, greatly promoted by the hurry of the people for lighter clothing; but it would be a great deal better to wear the entire winter suits through March, and even to the middle of April; and even then, until the first week in May, to make no change in the outer clothing, nor any in the inner garments, except to a less heavy woolen next the skin; for it is only for the three hours embracing one o'clock in the afternoon that winter clothing is at all oppressive; while the very warmth of noonday makes the raw dampness of the morning and late afternoons specially felt.

All changes to a lighter or cooler garment should be made at dressing in the morning, and if in any case the change leaves the body chilly, or soon after it is made the weather changes to be much cooler, by all means promptly, without half an hour's delay, resume the full winter dress. The old, the young, the invalid, in short all persons of feeble constitutions, of small vitality, should be especially careful to heed these suggestions; inattention to which gives rise to the very frequent announcements in the morning papers, in the early spring, "Died suddenly, yesterday, ———, of pneumonia," — often, the very friend whom we had met in the street, or at church, within a week, apparently as well and as hearty as ever before.

